# Metagenomic sequencing dataset of microbial communities in onion and cabbage microgreens across substrates, *Salmonella* inoculation, and bacteriophage application

**DOI:** 10.1016/j.dib.2025.112297

**Published:** 2025-11-20

**Authors:** Elijah Ayilaran, Olivia McHugh, Yangjin Jung

**Affiliations:** a Biotechnology Program, West Virginia State University, Institute, WV 25112, USA; b Agricultural & Environmental Research Station, West Virginia State University, Institute, WV 25112, USA

**Keywords:** Microgreens, Shotgun metagenomics, Bacteriophage, *Salmonella*, Substrate, Microbial community

## Abstract

This dataset comprises shotgun metagenomic sequencing results from edible portion of onion (*Allium cepa*) and cabbage (*Brassica oleracea*) microgreens cultivated on soil, biostrate, and jute fiber substrates, with and without *Salmonella* inoculation and bacteriophage application. Table 1 contains detailed sequencing quality metrics and National Center for Biotechnology Information Sequence Read Archive accession numbers (BioProject: PRJNA1327464) for all 24 samples. Figure 1 provides a species-level (≥5% relative abundance) heatmap highlighting microbial community clustering by seed type. These data can be reused for comparative microbiome analyses, evaluation of pathogen-phage-substrate interactions, and benchmarking of metagenomic workflows.

Specifications Table

**Table:** 

Subject	Biology
Specific subject area	Food microbiology, Metagenomics
Type of data	Figure, Table, and fastq files.
Data collection	Shotgun DNA sequencing using Element AVITI™ platform (2 × 150 bp). Taxonomic profiling was performed using CosmosID-HUB with Kepler™ multi-kingdom taxonomic profiler.
Data source location	Microgreen cultivation and DNA extraction were conducted at the Agricultural & Environmental Research Station, West Virginia State University (Institute, WV, USA). Extracted DNA samples were sent to CosmosID Inc. (Rockville, MD, USA) for library preparation, shotgun DNA sequencing, and taxonomic profiling.
Data accessibility	All raw sequencing data generated in this study have been deposited in the National Center for Biotechnology Information (NCBI) Sequence Read Archive (SRA) under BioProject accession number PRJNA1327464. Repository name: NCBI SRA Data identification number: PRJNA1327464 Direct URL to data: https://www.ncbi.nlm.nih.gov/bioproject/PRJNA1327464
Related research article	This Data in Brief article supports the research article entitled “E. Ayilaran, O. McHugh, and Y. Jung. Bacteriophage application to reduce Salmonella and its effect on microbial diversity in microgreen production. Food Control (2025). Under review”, which has been submitted to Food Control and is currently under peer review.

## 1 Value of the Data

• Provides species-level microbial profiles of onion and cabbage microgreens across substrates, inoculation, and phage treatments.
• Offers high-quality sequencing metrics and SRA accessions, enabling reproducibility and cross-study comparisons.
• Enables researchers to reuse abundance matrices for meta-analyses on plant-microbiome-pathogen interactions.
• Supports development of biocontrol strategies by linking bacteriophage treatments to microbial ecology outcomes.

## 2 Background

This dataset was compiled to address food safety challenges associated with microgreen cultivation, especially the risk of *Salmonella* contamination under warm and humid conditions. Since microgreens are primarily consumed raw, they lack a kill step, and conventional post-harvest interventions such as washing are unsuitable due to tissue fragility and reduced shelf life [1]. This indicates the need for effective pre-harvest strategies that can be integrated into cultivation without affecting microgreens' quality. Bacteriophages have emerged as a potential biocontrol option because of their specificity to bacterial hosts and minimal effect on product attributes. However, limited information is available on their use in microgreen cultivation and how their performance may be influenced by growing substrates, which are known to shape microbial communities and pathogen persistence [2,3]. To address this knowledge gap, the dataset was generated using shotgun metagenomic sequencing to document *Salmonella* levels and microbial diversity in onion and cabbage microgreens cultivated on soil, biostrate, and jute fiber, with and without bacteriophage application. This dataset complements an associated research article by providing raw sequencing data and detailed metadata, enabling secondary analyses, reproducibility, and comparative studies in food safety and microbial ecology.

## 3 Data Description

The dataset comprises raw shotgun metagenomic sequences and processed taxonomic profiles of the edible portions of onion (*Allium cepa*) and cabbage (*Brassica oleracea*) microgreens cultivated on different substrates under both non-*Salmonella*-inoculated and *Salmonella*-inoculated and treated with *Salmonella* specific bacteriophage. Raw sequence data in FASTQ format are available in the National Center for Biotechnology Information (NCBI) Sequence Read Archive (SRA) under BioProject accession number PRJNA1327464. Sequencing quality metrics and individual SRA accessions are summarized in Table 1, based on FastQC (v0.11.9) reports.

**Table 1:** Summary of sequencing quality metrics and SRA accession for onion microgreen samples across treatments and substrates.

Seed	Treatment	Substrate	Total Raw Reads	Unique Reads	% Unique Reads	Avg Phred score	GC Content (%)	SRA accession
ON	NoSal	Biostrate	23,957,310	21,052,326	87.9	39.4	59 %	SRX30448090
ON	NoSal	Jute Fiber	30,473,432	27,365,616	89.8	39.9	57 %	SRX30448094
ON	NoSal	Soil	25,643,536	23,538,172	91.8	39.7	57 %	SRX30448072
ON	Control	Biostrate	23,051,576	21,266,290	92.3	38.9	61 %	SRX30448091
ON	Control	Jute Fiber	30,699,928	26,339,375	85.8	39.6	58 %	SRX30448095
ON	Control	Soil	30,858,570	27,413,144	88.8	39.5	57 %	SRX30448073
ON	T1	Biostrate	26,009,694	22,856,472	87.9	39.3	59 %	SRX30448092
ON	T1	Jute Fiber	34,788,112	32,528,139	93.5	39.5	56 %	SRX30448074
ON	T1	Soil	34,975,662	31,190,268	89.2	39.7	57 %	SRX30448084
ON	T2	Biostrate	28,954,360	24,254,199	83.8	39.2	59 %	SRX30448093
ON	T2	Jute Fiber	41,811,174	35,872,520	85.8	39.2	60 %	SRX30448075
ON	T2	Soil	26,891,400	24,842,340	92.4	39.6	57 %	SRX30448089
CA	NoSal	Biostrate	35,660,542	29,840,367	83.7	39.7	57 %	SRX30448080
CA	NoSal	Jute Fiber	39,668,012	33,265,192	83.9	39.3	59 %	SRX30448085
CA	NoSal	Soil	47,145,060	34,629,051	73.5	39.5	59 %	SRX30448076
CA	Control	Biostrate	36,208,068	31,690,947	87.5	39.7	57 %	SRX30448081
CA	Control	Jute Fiber	74,925,766	58,451,733	78	39.6	58 %	SRX30448086
CA	Control	Soil	36,472,088	32,095,399	88	39.4	60 %	SRX30448077
CA	T1	Biostrate	35,803,020	29,391,033	82.1	39.7	56 %	SRX30448082
CA	T1	Jute Fiber	38,804,826	30,737,045	79.2	39.6	58 %	SRX30448087
CA	T1	Soil	37,080,224	33,691,918	90.9	39.4	59 %	SRX30448078
CA	T2	Biostrate	39,646,448	32,266,833	81.4	39.7	57 %	SRX30448083
CA	T2	Jute Fiber	41,186,506	35,440,915	86	39.5	57 %	SRX30448088
CA	T2	Soil	40,721,196	38,229,196	93.9	38.8	60 %	SRX30448079

Four treatment groups were included: (i) non-inoculated (NoSal), and three *Salmonella*-inoculated groups consisting of Control (no bacteriophage), T1 (bacteriophage applied immediately after blackout), and T2 (bacteriophage applied 24 h before harvest), applied across each growing substrate. In total, 861 million paired-end reads were generated across 24 samples, averaging 35.9 million reads per sample. After quality filtering, 86.5 % of reads were retained for downstream taxonomic and functional profiling. All raw reads were provided in standard FASTQ format with Sanger/Illumina 1.9 (Phred+33) quality encoding, and each sample exhibited an average Phred quality score greater than 38.

Taxonomic classification of quality-filtered reads identified 16 phyla, 196 families, 225 genera, and 830 species across all samples. The dominant phylum was Pseudomonadota (91 %), followed by Mucoromycota (5.65 %), Bacteroidota (0.65 %), Ascomycota (0.63 %), Bacillota (0.54 %), Actinomycetota (0.39 %), Basidiomycota (0.21 %), and Microsporidia (0.075 %), with the remainder (<1 %) distributed among other low-abundance phyla.

**Fig. 1:**
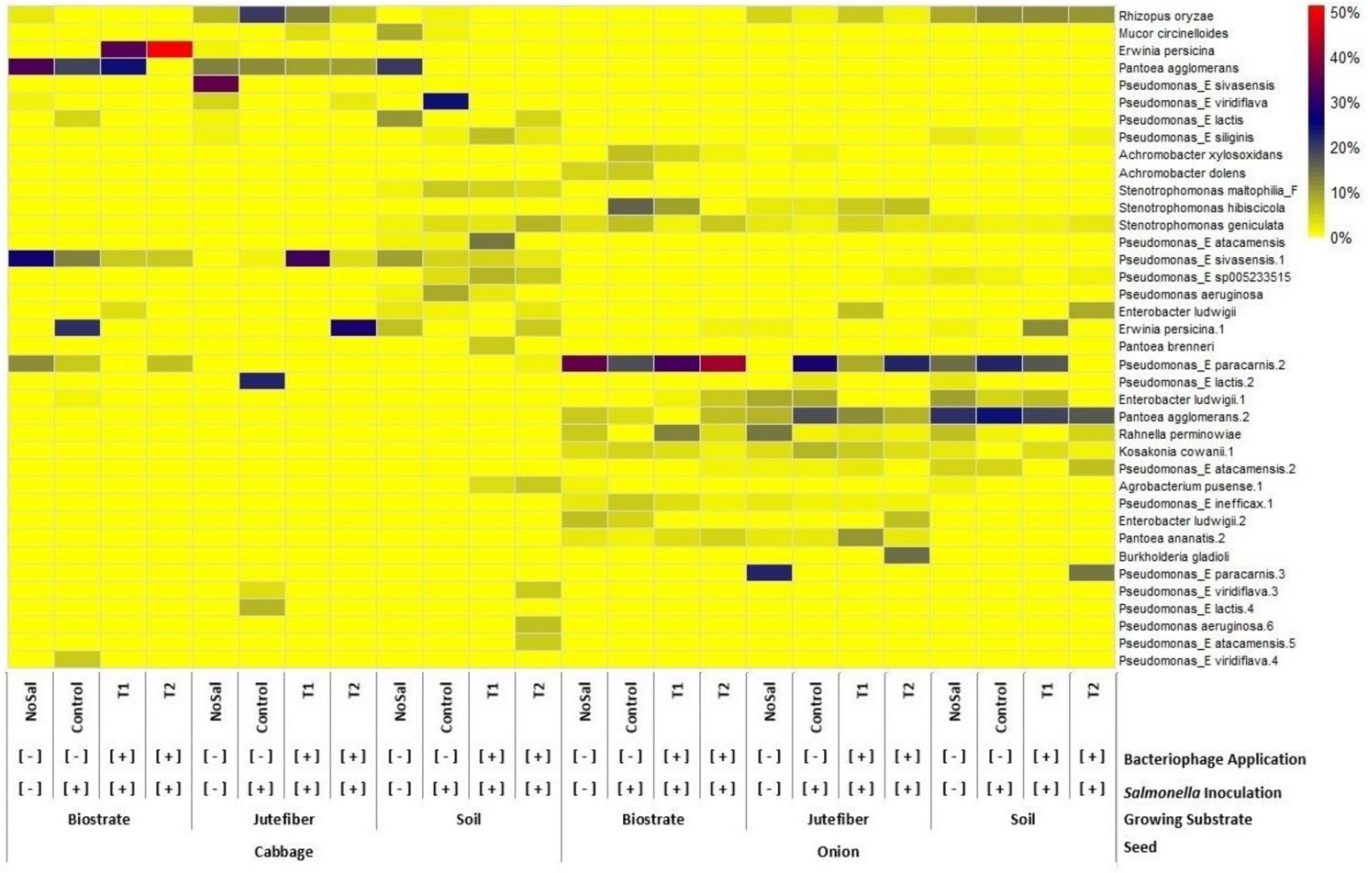
A Heatmap of microbial communities on the edible portions of onion and cabbage microgreens at species-level (≥5 %). The x-axis (top to bottom) indicates bacteriophage application (-/+), *Salmonella* inoculation (-/+), growing substrate (soil, biostrate, and jute fiber), and seed type (onion and cabbage).

Relative abundance profiles are organized by seed type (onion vs. cabbage), inoculation group (non-inoculated vs. *Salmonella*-inoculated), and bacteriophage treatment (Control, T1, T2), enabling comparisons of microbial community composition at the species level. As shown in Fig. 1, clustering patterns revealed distinct separation by seed type. *Pseudomonas_E paracarnis* predominated in onion samples, whereas *Pantoea agglomerans* was most abundant in cabbage, particularly when grown on jute and soil substrates. These species-level distributions highlight contrasting microbial community structures between onion and cabbage microgreens.

## 4 Experimental Design, Materials and Methods

### 4.1 Experimental design and sample preparation

**Fig. 2:**
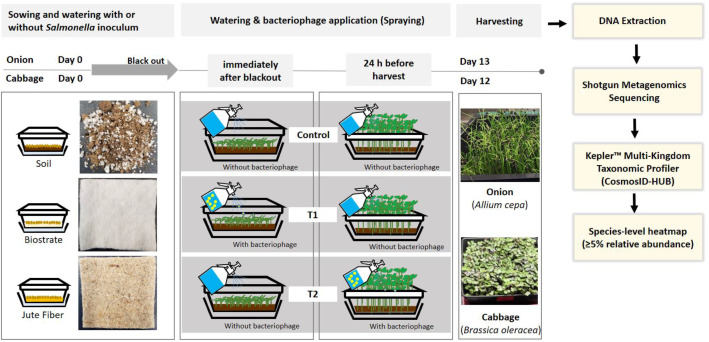
Schematic overview of experimental design.

As illustrated in Fig. 2, onion (*Allium cepa*) and cabbage (*Brassica oleracea*) microgreens were cultivated on three substrates (Biostrate, jute fiber, and soil-based) under four treatments: non-inoculated (NoSal) and *Salmonella*-inoculated groups, which included Control (no bacteriophage), T1 (bacteriophage applied immediately after blackout), and T2 (bacteriophage applied 24 h before harvest) [4]. PhageGuard S™ (Micreos, Wageningen, The Netherlands) was applied via spraying [5,6]. Edible portions of microgreens were harvested at day 13 for onion and day 12 for cabbage, and it DNA was extracted from the homogenate utilizing the DNeasy PowerSoil Pro Kit (QIAGEN, Hilden, Germany), following the guidelines provided by the manufacturer at the Agricultural & Environmental Research Station, West Virginia State University (Institute, WV, USA) [7]. The DNA concentrations were determined using the Quantus™ Fluorometer (Promega, Madison, WI, USA) after calibration. Subsequently, the extracted DNA samples were preserved at −80 °C before being shipped to CosmosID (CosmosID Inc., Rockville, MD) for or library preparation and sequencing.

### 4.2 DNA library and shotgun metagenomics sequencing

DNA libraries were prepared using the Watchmaker DNA Library Prep Kit (7K0019–1 K; Watchmaker Genomics, Boulder, CO, USA). Genomic DNA was fragmented with the Watchmaker Frag/AT Buffer and Enzyme Mix, followed by adapter ligation using IDT xGen stubby adapters and unique dual index (UDI) primers. Libraries were amplified by seven PCR cycles, purified with CleanNGS magnetic beads (CleanNA), and eluted in nuclease-free water. Quantification was performed using a Qubit™ fluorometer with the dsDNA HS Assay Kit. Libraries were circularized via the Element Adept compatibility workflow and sequenced on the Element AVITI™ platform (Element Biosciences, San Diego, CA, USA) using the AVITI 2 × 150 bp Cloudbreak sequencing kit.

### 4.3 Quality control, read processing, and taxonomic profiling

Raw FASTQ files were generated with standard Phred+33 encoding (Sanger/Illumina 1.9). Sequencing quality was assessed using FastQC (v0.11.9), which provided metrics for read length, total reads, percentage of unique reads, GC content, and Phred quality scores (Table 1). Filtered reads were analyzed on the CosmosID-HUB platform (CosmosID Inc., Rockville, MD, USA) using the Kepler™ multi-kingdom taxonomic profiler. This pipeline integrates (i) a curated multi-kingdom biomarker database (GenBook™), (ii) k-mer–based taxonomic classification, and (iii) probabilistic Smith–Waterman alignment for abundance estimation and refinement. Further details on the Kepler™ Host-Agnostic Taxonomic Profiling algorithms are available online (https://docs.cosmosid.com/docs/kepler-microbiome-profiler#pre-computational-stage-for-curating-and-building-a-comprehensive-biomarker-database-genbook**)**. Relative abundance data on taxonomic profiles from the kingdom to strain levels were retrieved. Species-level abundance data were exported from the CosmosID Hub as TSV files and imported into R using the *readxl* package. To highlight dominant taxa, a 5 % relative abundance threshold was applied, and the filtered data were visualized as a heatmap in R (v4.4.2; R Core Team, 2024) with the *pheatmap* and *ggplot2* packages [8].

## Limitations

Not applicable

## Ethics Statement

The study follows the ethical requirements for publication in *Data in Brief*. It does not involve human subjects, animal experiments, or any data collected from social media platforms.

## CRediT Author Statement

**Elijah Ayilaran:** Methodology, Investigation, Formal analysis, Writing – original draft; **Olivia McHugh:** Methodology, Investigation, Writing- review & editing; **Yangjin Jung:** Conceptualization, Methodology, Validation, Formal analysis, Resources, Visualization, Supervision, Project administration, Funding acquisition, Writing –original draft.

## Data Availability

NCBI SRAMicrobial Community Dynamics and Salmonella Persistence in Microgreen Cultivation: Impact of Growing Substrates and Phage Biocontrol (Original data).

## References

[1] Turner E.R., Luo Y., Buchanan R.L. (2020). Microgreen nutrition, food safety, and shelf life: a review. J. Food Sci..

[2] Du M., Xiao Z., Luo Y. (2022). Advances and emerging trends in cultivation substrates for growing sprouts and microgreens toward safe and sustainable agriculture. Curr. Opin. Food Sci..

[3] Wright K.M., Holden N.J. (2018). Quantification and colonisation dynamics of *Escherichia coli* O157:H7 inoculation of microgreens species and plant growth substrates. Int. J. Food Microbiol..

[4] Ayilaran, E., McHugh, O., and Jung, Y. (2025). Bacteriophage application to reduce *Salmonella* and its effect on microbial diversity in microgreen production. *Food Control*. Under review

[5] De Vegt B., Sirdesai S., Peterson R., Pinheiro M., Nuboer W., Kan A., Van Mierlo J. (2019). Efficieny of phage intervention against *Salmonella* in meat and poultry processing. Meat Muscle Biol..

[6] Marti R., Zurfluh K., Hagens S., Pianezzi J., Klumpp J., Loessner M.J. (2013). Long tail fibres of the novel broad-host-range T-even bacteriophage S16 specifically recognize *Salmonella* OmpC. Mol. Microbiol..

[7] Dong M., Feng H. (2022). Microbial community analysis and food safety practice survey-based hazard identification and risk assessment for controlled environment hydroponic/aquaponic farming systems. Front. Microbiol..

[8] Wen T., Niu G., Chen T., Shen Q., Yuan J., Liu Y.X. (2023). The best practice for microbiome analysis using R. Protein Cell.

